# Dulaglutide Ameliorates Intrauterine Adhesion by Suppressing Inflammation and Epithelial–Mesenchymal Transition via Inhibiting the TGF-β/Smad2 Signaling Pathway

**DOI:** 10.3390/ph16070964

**Published:** 2023-07-05

**Authors:** Yifan Wang, Yixiang Wang, Yang Wu, Yiqing Wang

**Affiliations:** 1 The First School of Clinical Medicine, Lanzhou University, Lanzhou 730000, China; yifanwang927@163.com (Y.W.); wangyixiang9511@163.com (Y.W.); wuyang21@lzu.edu.cn (Y.W.); 2 Gansu International Scientific and Technological Cooperation Base of Reproductive Medicine Transformation Application, Gansu Key Laboratory of Reproductive Medicine and Embryo, Lanzhou 730000, China

**Keywords:** dulaglutide, intrauterine adhesion, inflammation, endometrial fibrosis, epithelial–mesenchymal transition, TGF-β/Smad2

## Abstract

Intrauterine adhesion (IUA) is a common gynecological disease with limited therapeutic options. Dulaglutide is a long-acting glucagon-like peptide-1 (GLP-1) analog with some anti-fibrotic and anti-inflammatory properties; however, its action on IUA remains uncertain. The purpose of the experiments in this study was to explore the effect of dulaglutide on IUA and to elucidate its mechanism to provide new ideas for the clinical treatment of IUA. An IUA mouse model was established via mechanical curettage and inflammation induction; mice received subcutaneous injection with three doses of dulaglutide once a day for two weeks (treatment) or equal amounts of sterile ddH_2_O (control), and sham-operated mice were treated similarly to the control mice. Mice were sacrificed, and uterine tissues were subjected to hematoxylin and eosin (H&E) and Masson’s trichrome staining for histomorphological and pathological analyses and real-time quantitative polymerase chain reaction (RT-qPCR) and Western blotting (WB) for gene and protein expression analyses. Dulaglutide improved the shape of the uterine cavity, increased endometrial thickness and the number of glands, and significantly reduced the area of collagen fiber deposition in the endometrium. It significantly reduced collagen type I A 1 (COL1A1), interleukin-1beta (IL-1β), interleukin-6 (IL-6), tumor necrosis factor-alpha (TNF-α), C-C motif chemokine ligand 2 (CCL2), F4/80 (macrophage), vimentin and transforming growth factor-beta (TGF-β) mRNA levels and COL1A1, IL-1β, IL-6, TNF-α, F4/80, vimentin, E-cadherin, TGF-β, and p-Smad2 protein expression levels. This study demonstrates that dulaglutide reduces inflammatory responses by inhibiting M1 macrophage polarization and inflammatory factor release and may ameliorate fibrosis by inhibiting epithelial–mesenchymal transition (EMT) via TGF-β/Smad2 signaling.

## 1. Introduction

Intrauterine adhesion (IUA) is a common gynecological disease characterized by pathological changes involving inflammation and fibrinogen accumulation in the extracellular matrix, resulting in endometrial fibrosis and eventually leading to oligomenorrhea, amenorrhea, and infertility [1]. Although there are no clinical data on the prevalence of IUA in asymptomatic patients, the prevalence of IUA is as high as 21.5% in patients with a history of postpartum curettage and 19.1% after an abortion [2]. In addition, due to the rapid development of medical imaging and hysteroscopy, the diagnostic rate of IUA is increasing annually. Currently, hysteroscopic adhesiolysis is the treatment of choice for IUA; however, intrauterine devices, intrauterine support balloons, and biomaterials can be used to reduce IUA symptoms. Although progress has been made in hysteroscopic surgery and other treatment methods, there is still no effective method to treat moderate and severe IUA and prevent its recurrence. Therefore, it is necessary to explore the pathogenesis of IUA to develop new treatment methods.

IUA is characterized by endometrial fibrosis, which is one of the most serious complications in patients with injuries of the endometrial basal layer. Repetitive injuries to the endometrial basal layer result in the formation of scar tissues that can partially or completely obstruct the uterine cavity. Recently, epithelial–mesenchymal transition (EMT), one of the most important mechanisms of fibrotic diseases, has been shown to be intimately involved in the pathogenesis of endometrial fibrosis. EMT is a developmental process by which epithelial cells transition into mesenchymal cells and is widely present in the process of injury repair. In addition, it plays an important role in the fibrosis of multiple organs such as the liver, kidneys, lungs, and intestines [3,4,5,6]. EMT is characterized by the acquisition of a mesenchymal phenotype through inhibition of the components of the junctional complex, causing a loss of adhesion between epithelial cells, thereby enhancing the migration ability of cells. In addition, EMT plays a crucial role in the formation of organ damage and fibrosis caused by trauma [7]. In endometriosis, epithelial cells lose cell–cell adhesion and progress to a more mesenchymal phenotype [8]. Transforming growth factor β1 (TGF-β1), an archetypical pro-inflammation and fibrosis cytokine, is related to numerous biological processes, including inflammatory activity, cell adhesion, and EMT progress. In the injured endometrium of an IUA animal model, the mesenchymal marker vimentin was increased whereas the epithelial marker E-cadherin was decreased [9]. Guo et al. [10] provided specific evidence suggesting that TGF-β1/BMP7/Smad signaling was associated with EMT in a rat IUA model. In addition, Yao et al. [11] reported that bone marrow stem cell (BMSC)-derived exosomes can promote endometrium recovery by reversing EMT via targeting the TGF-β1/Smad pathway. Collectively, these previous findings suggest that EMT is likely to be one of the main mechanisms of impaired endometrial repair in IUA. Therefore, inhibiting EMT may be a novel strategy for the treatment of IUA.

Macrophages in uterine tissue are important inflammatory cells in the process of endometrial injury. In different microenvironments, macrophages are polarized into two distinct functional phenotypes; the M1 phenotype is classically activated, while the M2 phenotype is alternatively activated. M1 macrophages secrete interleukin (IL)-6 and tumor necrosis factor-alpha (TNF-α) in response to activation of toll-like receptors (TLRs) and cytokines, such as interferon-γ (IFN-γ), which lead to the processes of proinflammation and chemotaxis, thus inducing matrix degradation. In the early stage of uterine injury, M1 macrophage polarization and the subsequent release of inflammatory factors may be key factors for the induction of inflammatory injury [12,13]. Furthermore, there have been studies which demonstrated that the inhibition of M1 macrophage polarization accelerated fibrosis [14]. Thus, the downregulation of M1 macrophage polarization may be an effective strategy for the prevention and treatment of endometrial injury and fibrosis. Screening highly effective pharmaceutical agents that can inhibit the polarization of M1 macrophages provides an opportunity for the development of anti-inflammatory and anti-fibrotic drugs.

Glucagon-like peptide-1 (GLP-1) is a gastrointestinal hormone secreted by L cells in the terminal ileum and colon mucosa of the body. It exhibits important functions to stabilize blood sugar levels, including promoting insulin secretion, inhibiting glucagon secretion, and stimulating β cell proliferation and differentiation. GLP-1 is a suitable drug for the treatment of patients with type 2 diabetes (T_2_DM). However, natural GLP-1 has a short half-life (1–2 min), and when secreted into the bloodstream, it is rapidly degraded by dipeptidyl peptidase 4 (DPP-4), thereby losing its proinsulin secretion function [15]. Therefore, to improve the clinical application of GLP-1, drug development has focused on modifying the structure of GLP-1, retaining its biological effects by binding to the GLP-1 receptor while also making it less prone to rapid degradation by DPP-4 and extending its half-life to reach pharmacological concentrations. GLP-1 analogs, such as exenatide, liraglutide, dulaglutide, semaglutide, and lixisenatide, have been launched sequentially [16]. In addition to their hypoglycemic effects, GLP-1 analogs with long half-lives and lasting biological activities have been demonstrated to have many other biological effects, including cardiovascular and neuroprotective effects [17]. Various studies have shown that GLP-1 analogs can reduce tissue fibrosis and inflammatory response [18,19]. Li et al. demonstrated that liraglutide alleviates renal tubulointerstitial fibrosis induced by unilateral ureteral obstruction (UUO) by inhibiting the TGF-β/Smad3 and ERK1/2 signaling pathways [20]. Liraglutide alleviates cardiac fibrosis in various pathological conditions [21] and reduces renal inflammation and fibrosis in a rat model of diabetes [22]. In addition, recent studies have shown that lixisenatide has positive anti-inflammatory effects in the treatment of tissue fibrosis [23]. Based on these findings, we previously investigated the effects of the GLP-1 analog exenatide to treat IUA in mice and demonstrated that exenatide effectively ameliorates IUA [24]. However, we did not investigate which downstream signaling molecules were triggered by treatment with exenatide.

Dulaglutide, a long-acting GLP-1 receptor agonist (with a half-life of approximately 5 days) with 90% human homology, approved by the US FDA in 2014, is a novel hypoglycemic drug whose activity is mediated via the specific interaction between it and the GLP-1 receptor. Dulaglutide is a once-weekly GLP-1 receptor agonist that has been widely used to treat type 2 diabetes, and its convenience is very much what patients expect nowadays. Researchers [25] have demonstrated that dulaglutide can ameliorate renal fibrosis through suppressing EMT and the upstream TGF-β1/Smad signaling in rats. To the best of our knowledge, however, no report has yet examined the long-term efficacy of dulaglutide on IUA. Therefore, this experiment explores both the effect of single use of the dulaglutide on IUA-like mice and the underlying mechanism to provide new treatment methods and ideas for the clinical treatment of IUA.

## 2. Results

### 2.1. Dulaglutide Improves Endometrial Morphology and Reduces Collagen Fiber Deposition

**Figure 1:**
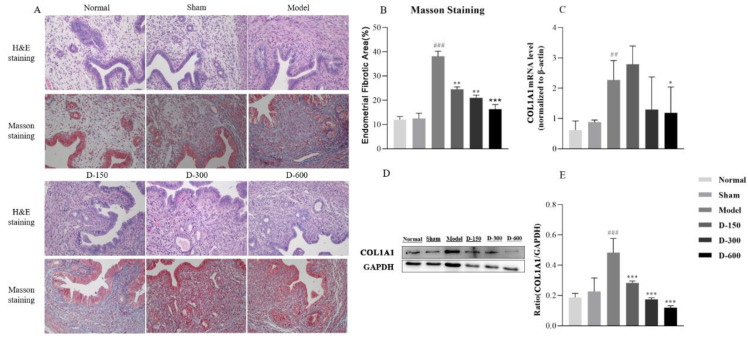
(**A**) Histological structures of the uterus in the six experimental groups (H&E and Masson’s trichrome staining). (**B**) Analysis of the fibrotic area in the endometrium in each group at 14 days after IUA model establishment. (**C**) RT-qPCR analysis of the relative mRNA expression levels of COL1A1 in each group. (**D**,**E**) Western blot analysis of the protein expression levels of COL1A1 in the uteri of mice in each group. Data are expressed as mean ± SD. ## *p* < 0.01, ### *p* < 0.001 vs. normal group; * *p* < 0.05, ** *p* < 0.01, and *** *p* < 0.001 vs. model group.

The surface of the uterine cavity of mice in the normal and sham groups was smooth, the structure was complete, and the glands were abundant. In the IUA model group, the thickness of the endometrium was different; adhesions or even atresia appeared in the uterine cavity, the glands were damaged and atrophied, and the number of glands was significantly reduced. After 2 weeks of treatment with different doses of dulaglutide, the shape of the uterine cavity was improved and endometrial thickness and the number of glands were increased (Figure 1A).

Masson’s trichrome staining revealed that mice in the normal and sham groups had low amounts of endometrial collagen fibers whereas numerous blue-stained collagen fibers were observed in the IUA model group, accompanied by an increase in new blood vessels, with some IUA mice showing adhesion and occlusion in the uterine cavity. After 14 days of treatment with dulaglutide, the area of collagen fiber deposition in the endometrial stroma was significantly reduced compared to that in the IUA group (Figure 1A,B).

The mRNA and protein levels of COL1A1 were significantly increased in the model group (*p* < 0.01). After treatment with dulaglutide, COL1A1 mRNA expression was decreased, albeit not to a statistically significant degree, whereas COL1A1 protein expression was significantly decreased (*p* < 0.001) (Figure 1C–E).

### 2.2. Dulaglutide May Reduce Inflammatory Responses by Inhibiting M1 Macrophage Polarization and the Release of Inflammatory Factors

**Figure 2:**
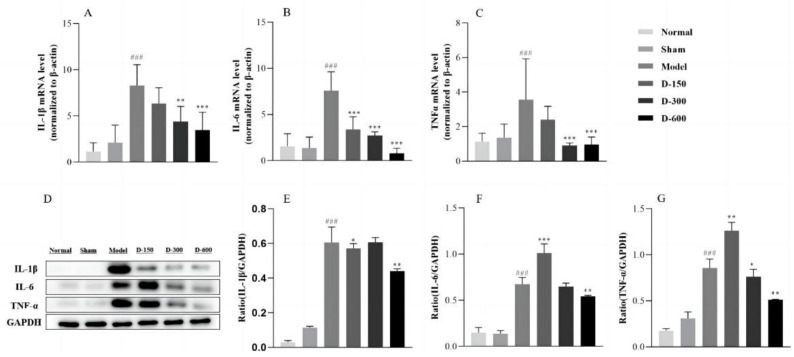
(**A**–**C**) mRNA expression of IL-1β, IL-6, and TNF-α in each experimental group according to RT-qPCR analysis. (**D**–**G**) Western blot analysis of the expression of IL-1β, IL-6, and TNF-α in the uteri of mice in each group. Data are expressed as the mean ± SD. ### *p* < 0.001 vs. normal group; * *p* < 0.05, ** *p* < 0.01, and *** *p* < 0.001 vs. model group.

**Figure 3:**
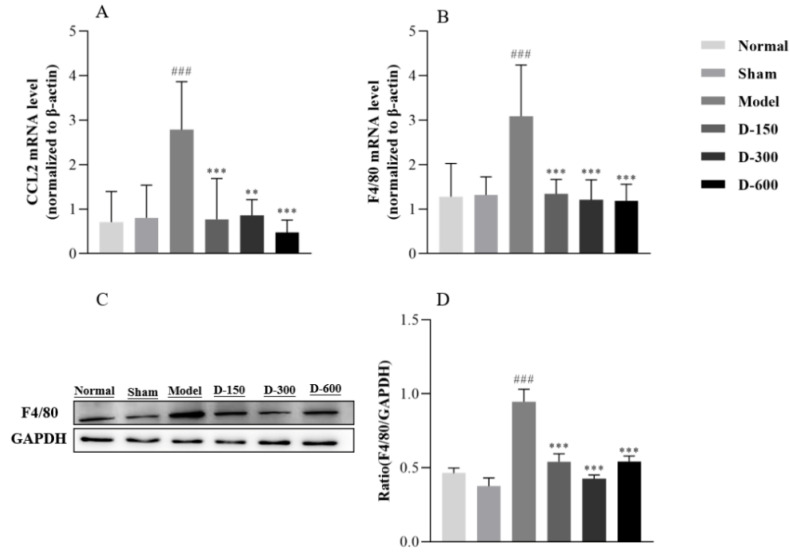
(**A**,**B**) RT-qPCR analysis of the relative mRNA expression levels of CCL2 and ADGRE1 in each experimental group. (**C**,**D**) Western blot analysis of the protein expression level of F4/80 in the uteri of the mice in each group. Data are expressed as mean ± SD. ### *p* < 0.001 vs. normal group; ** *p* < 0.01, and *** *p* < 0.001 vs. model group.

Compared to those in the control group, the mRNA and protein levels of IL-1β, IL-6, TNF-α, chemokine ligand 2 (CCL2), and F4/80 in the sham group showed no significant changes, whereas in the IUA group, they were significantly increased (*p* < 0.001) (Figure 2A–C and Figure 3A,B). After treatment with dulaglutide, the mRNA levels of IL-1β, IL-6, TNF-α, CCL2, and ADGRE1 (encoding F4/80) significantly decreased in a dose-dependent manner. The protein levels of the above-mentioned indicators were significantly decreased in the treated groups compared to those in the model group (*p* < 0.01) (Figure 2D–G and Figure 3C,D), except for the protein expression levels of IL-6 and TNF-α in the low-dose D-150 group, which were significantly increased (*p* < 0.01) (Figure 2F,G).

### 2.3. Dulaglutide May Ameliorate Fibrosis by Inhibiting Epithelial–Mesenchymal Transition via the TGF-β/Smad2 Signaling Pathway

**Figure 4:**
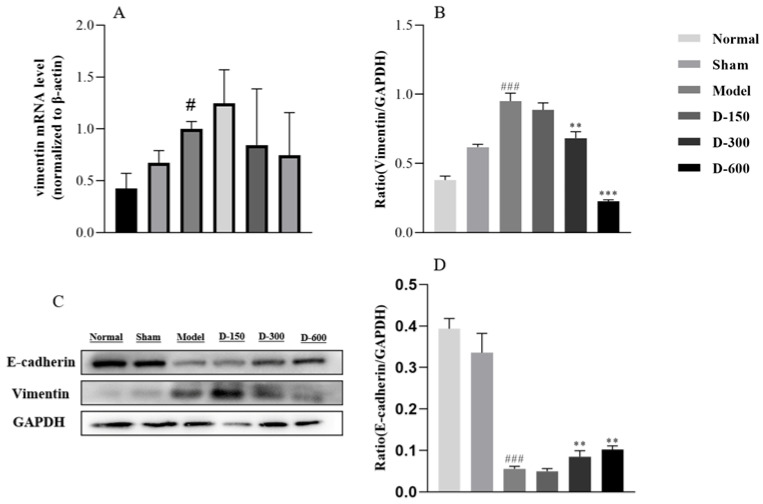
(**A**) RT-qPCR analysis of the relative mRNA expression level of VIM in each experimental group. (**B**–**D**) Western blot analysis of the protein expression levels of vimentin and E-cadherin in the uteri of the mice in each group. Data are expressed as mean ± SD. # *p* < 0.05, ### *p* < 0.001 vs. normal group; ** *p* < 0.01, and *** *p* < 0.001 vs. model group.

The relative VIM mRNA expression levels in the IUA group were increased compared to those in the normal and sham groups. After 14 days of treatment with medium and high doses of dulaglutide, relative VIM mRNA expression was reduced in a dose-dependent manner (Figure 4A). Compared to the levels in normal endometrium, the expression of E-cadherin was reduced and that of vimentin was increased in the IUA model group. After treatment with medium-dose (D-300) and high-dose (D-600) dulaglutide, E-cadherin expression was significantly increased (Figure 4B–D).

**Figure 5:**
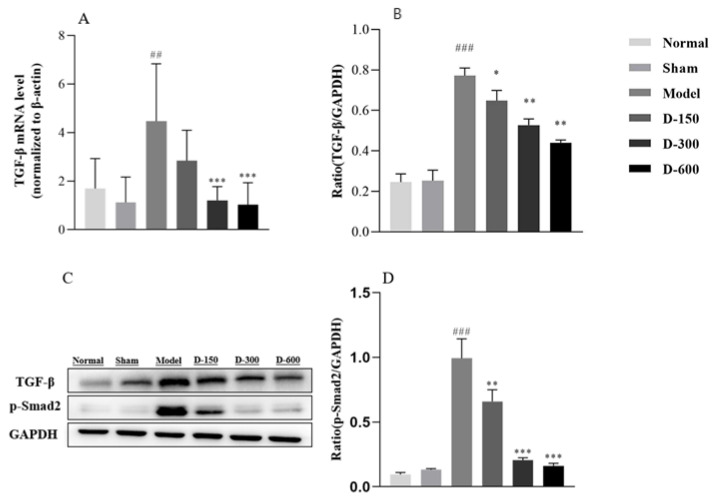
(**A**) RT-qPCR analysis of the relative mRNA expression level of TGF-β in each experimental group. (**B**–**D**) Western blot analysis of the protein expression levels of TGF-β and p-Smad2 in the uteri of the mice in each group. Data are expressed as mean ± SD. ## *p* < 0.01, ### *p* < 0.001 vs. normal group. * *p* < 0.05, ** *p* < 0.01, and *** *p* < 0.001 vs. model group.

In addition, we detected the levels of TGF-β and its key downstream molecule Smad2. The mRNA and protein levels of TGF-β and phosphorylated Smad2 in the model group were significantly increased, whereas they were generally significantly decreased after treatment with dulaglutide (Figure 5).

## 3. Discussion

IUA is a major health problem that causes medical issues, such as female infertility, irregular menstruation, and repeated abortions. Unfortunately, there is currently no effective strategy for treating IUA. Increased evidence suggests that endometrial fibrosis is associated with the development of IUA, but there is no existing satisfactory progress in potential treatment options. Therefore, more studies are needed to elucidate the mechanisms of endometrial fibrosis and to develop new prevention and treatment strategies.

Dulaglutide has been shown to inhibit fibroblast proliferation and ultimately prevent adhesion formation. However, the effect of dulaglutide on IUA remained unclear. Therefore, in our study, we successfully constructed a mouse IUA model through scraping injury and LPS-induced inflammation [24], as evidenced by glandular loss and increased levels of fibrosis (Figure 1), and our findings revealed the effect of dulaglutide on endometrial fibrosis for IUA. The results indicated that after treatment with dulaglutide, the number of glands was increased and collagen fiber deposition and COL1A1 mRNA and protein levels were reduced, which demonstrated that GLP-1 analogs have a preventive effect on IUA.

The occurrence and development of fibrosis is a complex pathological process, and research has shown that it is closely related to EMT, inflammatory response, cell apoptosis, and oxidative stress [26,27,28]. Infection and inflammatory exudates following endometrial damage are considered important risk factors for the development of IUA [1]. Inflammation, tissue formation, and tissue reconstruction are important processes in IUA fibrosis repair. Studies have revealed that when the basal layer is severely damaged, the endometrium loses its ability to repair and regenerate and numerous fibroblasts and inflammatory cells accumulate in the damaged endometrium, inducing an inflammatory response and secreting a wide range of inflammatory mediators [29]. In particular, they secrete TNF-α, IL-1β, and IL-6, which in turn promote the development of the disease [30,31]. Inflammation is mediated by many upstream and downstream molecules. Among them, GLP-1/GLP-1 receptor signals have been investigated as treatment targets for inflammation-related diseases, given their anti-inflammatory functions [32,33]. To this end, Chen et al. suggested that activation of the GLP-1 receptor with liraglutide could inhibit inflammation by decreasing the release of inflammatory mediators and regulating phosphoinositide 3-kinase (PI3K)/AKT signaling [34]. Xin Li et al. [35] reported that lixisenatide has a beneficial protective effect against multiple aspects of osteoarthritis (OA), including oxidative stress, expression of proinflammatory cytokines, and activation of the NF-κB proinflammatory signaling pathway. GLP-1 is known to improve insulin sensitivity and may decrease macrophage infiltration and suppress the inflammatory response. Previous studies have demonstrated that GLP-1 reduces the accumulation of monocytes/macrophages and the expression of inflammatory mediators such as TNF-α and monocyte chemotactic protein in activated macrophages [36]. In the present study, our experimental results showed that the mRNA and protein levels of TNF-α, IL-1β, and IL-6 were indeed significantly increased in the endometrium of IUA mice and their levels were significantly decreased after treatment with dulaglutide (Figure 2). This result suggests that dulaglutide has a protective effect against IUA and can reduce the release of pro-inflammatory factors after endometrial injury. Wang et al. [37] found that exendin-4 reduced liver lipids and macrophage contents as well as inflammation in mice with nonalcoholic steatohepatitis. Lu et al. [38] reported that the biological effects of macrophages on renal injury are related to the polarization of pro-inflammatory M1 macrophages or anti-inflammatory M2 macrophages, which are involved in inflammatory injury and renal tissue repair, respectively. Our present study revealed that the mRNA levels of the genes encoding regulatory proteins CCL2 and F4/80 related to M1 macrophage polarization were significantly increased in the IUA endometrium compared to their levels in the normal group. Treatment with dulaglutide for two weeks significantly reduced the expression levels of CCL2 and F4/80 in IUA mice (Figure 3). These results suggest that dulaglutide inhibits M1 macrophage polarization during endometrial injury treatment. Based on these experimental results, we speculate that dulaglutide alleviates inflammation by inhibiting the release of inflammatory factors and M1 macrophage polarization, which provides a new anti-inflammatory mechanism of GLP-1.

Although the mechanism of IUA remains unclear, accumulating evidence suggests that EMT plays a significant role in the process of endometrial fibrosis. After IUA endometrial injury, the expression of fibrosis markers and the mesenchymal markers N-cadherin and vimentin increases, whereas that of the epithelial marker E-cadherin decreases [39]. Zhou et al. [40] demonstrated that the severity of endometrial fibrosis in IUA mice is associated with increased expression of EMT-related proteins. In the present study, we found that the relative mRNA expression of vimentin, an EMT marker, was increased in the IUA model group, whereas it was inhibited after treatment with medium and high doses of dulaglutide. We further explored the expression of EMT-related proteins and found that the expression of E-cadherin was low, whereas that of vimentin was high, in the IUA model group. After treatment with dulaglutide, E-cadherin expression was significantly increased whereas vimentin expression was inhibited.

TGF-β plays a key role in wound healing and fibrogenesis and has been extensively studied in various models of fibrosis [41,42]. In fibrotic diseases, activated TGF-β regulates the fibroblast phenotype and function and induces myofibroblast trans-differentiation. In addition, TGF-β can induce EMT, promote the aggregation of fibroblasts and inflammatory cells and collagen synthesis, induce the synthesis of the extracellular matrix, and inhibit matrix degradation, further promoting fibrosis and tissue repair [43]. Bai et al. [44] reported that resveratrol antagonizes the Hedgehog signaling pathway in vivo and in vitro to inhibit EMT, thereby reducing the expression of TGF-β1 and inhibiting renal fibrosis. MicroRNA-29b mediates lung EMT and prevents pulmonary fibrosis [45]. TGF-β1 has been widely used to induce fibrosis and EMT in vitro [46]. In addition, microRNA-326 and microRNA-29b ameliorate fibrosis in human endometrial stromal cells by mediating the TGF-β1/Smad signaling pathway [47,48]. Elevated TGF-β expression in endometrial tissue of patients with IUA or animal models of IUA has been frequently reported, and it is positively correlated with the degree of adhesion [49]. In human endometrial epithelial cells, researchers have utilized TGF-β to induce EMT and successfully constructed an IUA cell model to explore potential therapeutic options for IUA in vitro [50]. Bao et al. [51] demonstrated that TGF-β1/Smad signaling coincides with EMT in a rat model of IUA. Our study revealed that TGF-β1 and p-Smad 2 protein expression was significantly increased in the IUA endometrium. After treatment with dulaglutide, TGF-β1 and p-Smad 2 levels were decreased in a dose-dependent manner. This result suggests that dulaglutide inhibits TGF-β1/Smad 2 signaling activation. Based on these experimental results, we speculate that the anti-fibrotic effect of dulaglutide may be due to its interference with EMT induced by the TGF-β/Smad2 signaling pathway.

It should be noted that our study was based on previous research and that the TGF-β/Smad signaling pathway and related molecules were detected after GLP-1 analog treatment. However, the relationship between GLP-1 analogs and the TGF-β signaling pathway requires further research and experimental validation. Nevertheless, this study provided preliminary insights into whether dulaglutide can suppress M1 macrophage polarization in the IUA endometrium. Further studies are warranted to clarify the connection between GLP-1 analogs and macrophages.

## 4. Methods

### 4.1. Animals and Chemicals

Thirty-six eight-week-old female C57BL/6J mice weighing 16–18 g were purchased from Beijing Vital River Laboratory Animal Technology Co., Ltd (Beijing, China). The mice were maintained and housed in a controlled environment for one week at a temperature of 25 °C and a light cycle of 12 h light/12 h dark. The animals were given free access to standard laboratory chow and water. All animal management and experimental procedures were performed in accordance with the Guidelines for the Care and Use of Laboratory Animals; the study was conducted in accordance with the Declaration of Helsinki and was approved by the Ethics Committee of the First Hospital of Lanzhou University (LDYYLL2022-433) for studies involving humans and animals.

Dulaglutide (98% purity) was purchased from GL Biochem (Shanghai, China).

### 4.2. Animal Model and Experimental Groups

The 36 mice were evenly divided into six groups: a normal control group (normal), a sham operation group (sham), an IUA + double-distilled H_2_O (H_2_O) group (model), an IUA + 150 μg/kg dulaglutide group (D-150), an IUA + 300 μg/kg dulaglutide group (D-300), and an IUA + 600 μg/kg dulaglutide group (D-600). To establish an IUA model, mechanical injury of the uterus was performed at the diestrus stage. The mice were anesthetized by intraperitoneal injection of pentobarbital sodium. The uterus was exposed by an excision in the low midline abdomen. IUA was induced by double injury (scraping injury and lipopolysaccharide (LPS)-induced inflammation), as previously described [24]. Subsequently, the abdominal cavity was closed. The surgical procedure was performed under sterile conditions. The animals in the sham operation group received identical treatment to those in the IUA group, with the exception of not undergoing uterine surgery. The animals in the normal group did not undergo any intervention. Mice in the IUA + dulaglutide groups were subcutaneously injected with 150, 300, or 600 μg/kg (dissolved in sterile ddH_2_O) once a week for two weeks, and mice in the IUA + ddH_2_O group were injected with equal amounts of sterile ddH_2_O. The route of administration was subcutaneous injection. On the 14th day after treatment, the mice were sacrificed via overdose from 4% chloral hydrate and their uteri were harvested.

### 4.3. Histopathological Evaluation

The uteri were fixed with 4% paraformaldehyde, embedded in paraffin, cut into 4 μm-thick sections, and subjected to hematoxylin and eosin (H&E) and Masson’s trichrome staining. All sections were photographed using an OCULAR camera-mounted Olympus BX73 microscope (Olympus Optical Co., Tokyo, Japan).

Under a 20’ magnification, the glands in the submucosa and basal layer of the endometrium were counted in three randomly chosen fields of view in each section stained with H&E, and the average value was calculated. Paraffin sections of uterine tissue were stained with Masson’s trichrome and collagen fibers stained blue. The degree of endometrial fibrosis was assessed by quantifying three random fields of view in each section stained with Masson’s trichrome. The positive stained area (blue pixels) of collagen was quantified with Image-Pro Plus 6.0 software, and the percentage of positive stained collagen area against total stained area was calculated.

### 4.4. RNA Extraction and Quantitative Reverse Transcription Polymerase Chain Reaction (RT-qPCR)

The uterine tissue samples were immediately placed in liquid nitrogen and stored at −80 °C for RT-qPCR. Frozen tissues from −80 °C were immediately homogenized in Trizol reagent according to the manufacturer’s instructions (Thermo Fisher, MA, USA). The integrity and concentration of the RNA were measured using a NanoDrop Spectrophotometer 1000 (Thermo Fisher Scientific, Waltham, MA, USA). cDNA was synthesized using the RevertAid First Strand cDNA Synthesis Kit (Yeasen, Shanghai, China). Target gene expression was evaluated by RT-qPCR on a 700 Fast Real-Time PCR System (Bio-Rad Laboratories, Hercules, CA, USA). The primers used are listed in Table 1. The PCR cycling parameters were as follows: 95 °C for 10 s, followed by 40 cycles of 95 °C for 30 s, 55 °C for 30 s, and 72 °C for 30 s. Relative mRNA expression levels were determined using the comparative Ct (ΔΔCt) method.

### 4.5. Western Blotting

The uterine tissues were frozen in liquid nitrogen and stored at −80 °C since collection was cut into small pieces and homogenized in RIPA buffer in a Dounce homogenizer. Total protein was briefly sonicated, incubated on ice for 30 min, and centrifuged at 12,000× *g* for 15 min. Supernatants were collected and stored at −80 °C until use. The protein concentrations were determined using a bicinchoninic assay protein assay kit (Thermo Fisher Scientific, Waltham, MA, USA). Samples containing 50 μg of protein were separated by 10% sodium dodecyl sulfate-polyacrylamide gel electrophoresis and transferred to a polyvinylidene difluoride membrane (Merck Millipore, Darmstadt, Germany). After being blocked with Tris-buffered saline with 0.1% Tween-20 (TBST) containing 5% nonfat powdered milk, the membrane was incubated with primary antibodies overnight at 4 °C using appropriate dilutions. After washing with TBST, the membrane was incubated with a horseradish peroxidase-conjugated anti-rabbit or anti-mouse IgG secondary antibody (Table 2). Signals generated by enhanced chemiluminescence (Saiguo Biotech, Co., Ltd., Guangzhou, China) were recorded using a Tanon-5200 Multi-imaging system. Protein expression levels were quantified using Image J software version 8.0 and normalized to the level of GAPDH.

### 4.6. Statistical Analysis

All data are presented as the mean ± standard deviation (SD). Results were analyzed by one-way analysis of variance (ANOVA) using GraphPad Prism version 9. Differences were considered statistically significant at *p* < 0.05.

## 5. Conclusions

In summary, in the present study, a mouse model of IUA was successfully established by scrape injury and LPS-induced inflammation. Moreover, we demonstrated that the GLP-1 analog dulaglutide exerts anti-fibrotic effects in IUA mice. We found that dulaglutide may reduce inflammatory responses by inhibiting M1 macrophage polarization and the release of inflammatory factors. Also, dulaglutide may ameliorate fibrosis by inhibiting EMT via the TGF-β/Smad2 signaling pathway. We speculate that applications of dulaglutide in patients with IUA will be possible in the future. Therefore, our results reveal that dulaglutide is a promising preventive agent for IUA. Certainly, further studies are warranted to explore the other possible mechanisms that may support the endometrium-protective effect of dulaglutide.

## Figures and Tables

**Table 1:** Primers used for quantitative reverse transcription polymerase chain reaction.

Gene	Forward Primer (5′-3′)	Reverse Primer (5′-3′)
TGF-β	CTCCCGTGGCTTCTAGTGC	GCCTTAGTTTGGACAGGATCTG
IL-1β	GCAACTGTTCCTGAACTCAACT	ATCTTTTGGGGTCCGTCAACT
IL-6	CCAAGAGGTGAGTGCTTCCC	CTGTTGTTCAGACTCTCTCCCT
TNF-α	CCCTCACACTCAGATCATCTTCT	GCTACGACGTGGGCTACAG
ADGRE1	TTGTACGTGCAACTCAGGACT	CCACGTCTCACCATTGGGG
COL1A1	GCTCCTCTTAGGGGCCACT	GATCCCAGAGTGTTGATGCAA
FN1	TTCAAGTGTGATCCCCATGAAG	CAGGTCTACGGCAGTTGTCA
VIM	CGGCTGCGAGAGAAATTGC	CCACTTTCCGTTCAAGGTCAAG
β-actin	CTGAGAGGGAAATCGTGCGT	TGTTGGCATAGAGGTCTTTACGG

**Table 2:** Primary and secondary antibodies used for Western blotting.

Antibody	Host	Dilution	Company	Catalog No.
Anti-TGF-β	Rabbit	1:1000	Cell Signaling Technology (MA, USA)	#3709
Anti-p-Smad2	Rabbit	1:2000	Cell Signaling Technology (MA, USA)	#8828
Anti-COL1A1	Rabbit	1:1000	Cell Signaling Technology (MA, USA)	#72026
Anti-E-cadherin	Mouse	1:1000	Cell Signaling Technology (MA, USA)	#14472
Anti-vimentin	Mouse	1:1000	Cell Signaling Technology (MA, USA)	#5741
Anti-F4/80	Mouse	1:1000	Bioss (Beijing, China)	bs-11182R
Anti-TNF-α	Mouse	1:1000	Cell Signaling Technology (MA, USA)	#11948
Anti-IL-1β	Mouse	1:200	Santa Cruz (Dallas, USA)	sc-12742
Anti-IL-6	Mouse	1:1000	Cell Signaling Technology (MA, USA)	#12912
Anti-GAPDH mouse monoclonal antibody	Mouse	1:5000	TransGene Biotech (Beijing, China)	HC301-01
Goat-anti-mouse IgG	Goat	1:10,000	OriGene (Wuxi, China)	ZB-2305
Goat-anti-rabbit IgG	Goat	1:10,000	OriGene (Wuxi, China)	ZB-2301

## Data Availability

All data are contained within the article.
